# Increased physiological dead space at exercise is a marker of mild pulmonary or cardiovascular disease in dyspneic subjects

**DOI:** 10.1080/20018525.2018.1492842

**Published:** 2018-07-05

**Authors:** Laurent Plantier, Christophe Delclaux

**Affiliations:** a INSERM UMR 1152, Labex Inflamex, Paris, France; b Université Paris Diderot, PRES Sorbonne Paris Cité, Paris, France; c Assistance-Publique-Hôpitaux de Paris, Hôpital Bichat-Claude Bernard, Service de Physiologie-Explorations Fonctionnelles, Paris, France; d Assistance-Publique-Hôpitaux de Paris, Hôpital Robert Debré, Service de Physiologie Pédiatrique, Paris, France

**Keywords:** Dead space, efficiency, exercise, cardiopulmonary exercise testing, lung disease, cardiovascular disease

## Abstract

**Background**: The characteristics of cardiopulmonary exercise testing (CPET)-derived parameters for the differential diagnosis of exertional dyspnea are not well known.

**Objectives:** We hypothesized that increased physiological dead space ventilation (VD/Vt) is a marker for mild pulmonary or cardiovascular disease in patients with exertional dyspnea.

**Design:** We used receiver operating characteristic analysis to determine the performance of individual CPET parameters for identifying subjects with either mild pulmonary or cardiovascular disease, among 77 subjects with mild-to-moderate exertional dyspnea (modified Medical Research Council scale 1–2).

**Results**: In comparison with subjects without disease, subjects with pulmonary disease (*n* = 31) had higher VE/V′CO_2_ slope, higher VD/Vt, and lower ventilatory reserve. Subjects with cardiovascular disease (*n* = 14) had lower heart rate and cardiovascular double product and higher VD/Vt at peak exercise. At a threshold of 28%, the sensitivity and specificity of VD/Vt at peak exercise for identifying pulmonary or cardiovascular disease were 89% (95% CI: 64–98%) and 72% (95% CI: 46–89%), respectively.

**Conclusions**: Increased physiological VD/Vt at exercise is a sensitive and specific marker of mild pulmonary or cardiovascular disease in dyspneic subjects.

## Introduction

Cardiopulmonary exercise testing (CPET) is a multicomponent procedure exploring respiratory, circulatory, and metabolic responses to exercise by integrating spirometry, gas exchange data, electrocardiographic and blood pressure monitoring, and arterial blood gas analysis in the course of incremental exercise [1]. CPET has been key to understanding mechanisms of exertional limitation in conditions affecting diverse systems [2–4]. Clinical applications of CPET are manifold and include prognostic evaluation of subjects with heart or lung disease [2,5].

**Figure 1.**
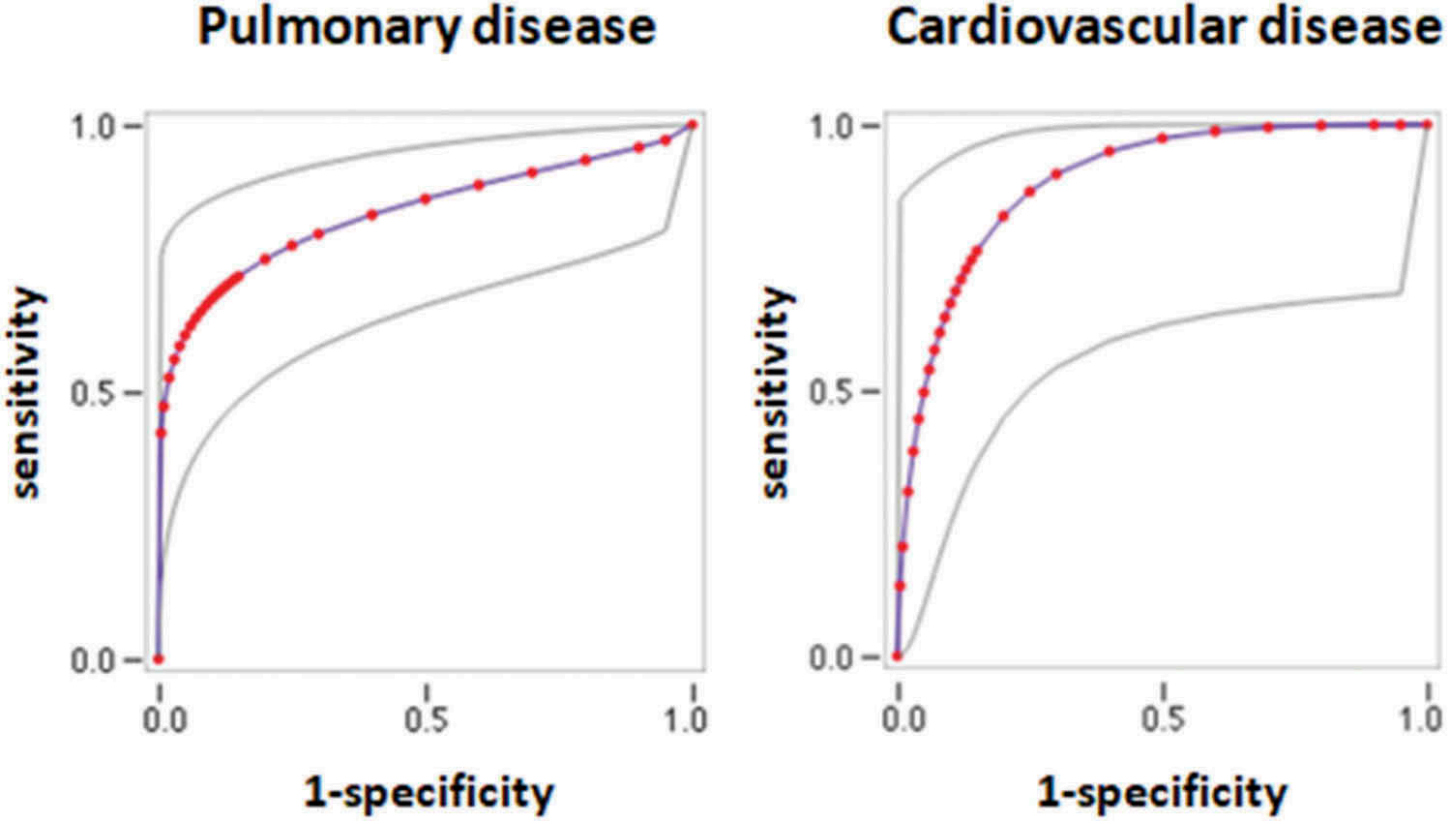
ROC curves of physiological VD/Vt for the diagnosis of pulmonary (left panel) or cardiovascular disease (right panel) disease in dyspneic subjects. Gray lines: upper and lower 95% confidence intervals.

CPET has an important role for the diagnostic evaluation of subjects with exertional limitation or dyspnea [1,6]. In this context, CPET may contribute either by affirming a specific diagnosis or, more commonly, by narrowing the differential diagnosis through the identification of features indicative of respiratory or circulatory dysfunction. In particular, in subjects without previously known disease and in whom non-invasive first-line tests fail to show severe cardiopulmonary disease which may explain moderate exertional dyspnea, CPET is a useful tool to identify whether exercise limitation or symptoms are associated with abnormalities in the oxygen and carbon dioxide transport pathway, mandating further testing, or the lack thereof [7], allowing to reassure the patient.

Interpretation of CPET results is commonly performed with the help of diagnostic algorithms or patterns such as those proposed by Wasserman et al. [8], the American Thoracic Society and American College of Chest Physicians [1], or the American Heart Association [6]. However, clinical validation of such algorithms is lacking, possibly due to the complex integrative nature of CPET and to the wide range of possible diagnoses. For instance, the classic Wassermann algorithms have a sensitivity of 79% and a specificity of 75% against an invasive hemodynamic gold standard for the diagnosis of a pulmonary vascular limitation [9], encouraging further study into the diagnostic performance of CPET. Recently, a reductionist approach was adopted, and studies were dedicated to determine the diagnostic performance of individual CPET parameters. A two-variable model, based on the CPET variables, oxygen pulse, and the ratio of VO_2_ to work rate, has a sensitivity of 87% and a specificity of 74% for the diagnosis of ischemic heart disease [10]. The difference between capillary PCO_2_ and end-expiratory PCO_2_, an indicator of dead space ventilation, is a sensitive and specific index for the diagnosis of chronic thromboembolic hypertension [11].

Because altered ventilation/perfusion matching is associated with both cardiac and lung diseases [12], we explored the utility of CPET parameters related to this concept for excluding pulmonary or cardiovascular disease. Specifically, we tested the hypothesis that increased physiological dead space ventilation would provide sensitive markers for the presence of disease affecting the respiratory or circulatory systems in subjects presenting with mild-to-moderate dyspnea, in a real-life clinical setting.

## Methods

### Study design

The study followed a retrospective case-control design. Subjects referred to the Physiology Department of a tertiary care hospital for the exploration of mild-to-moderate exertional dyspnea (modified Medical Research Council (mMRC) scale 1 or 2) over a 2-year period were considered for inclusion into the study. Patients referred for prognostic or preoperative evaluation were not included. Patients with a history of pulmonary or cardiovascular disease were included into the study if disease was either mild or well controlled, and it did not explain dyspnea according to the referring physician. Subjects with muscular, skeletal, or neurological conditions potentially contributing to exercise limitation, subjects with a World Health Organization performance status of grade 2 (unable to carry out any work activity) or more, subjects with a history of either lung or heart transplantation, and subjects with severely altered lung function (FEV1 < 60% predicted) were not included into the study.

Data were obtained from inpatient and outpatient chart review. All subjects had undergone blood cell counts ruling out anemia, lung function testing, chest X-rays or computed tomography, and echocardiography less than 6 months prior to referral. Subjects were allocated into three groups according to the existence of pulmonary or cardiovascular disease diagnosed either before or after CPET was performed. Subjects were allocated to the ‘No Disease’ group if all following criteria were met: (1) no prior history of chronic lung disease including asthma, (2) no prior history of heart failure, valvular disease, coronary heart disease, dysrhythmias, or resistant hypertension [13], (3) normal echocardiography, (4) normal lung function tests including spirometry, carbon monoxide diffusion capacity (DLCO), and arterial blood gases at rest, and (5) no clinical diagnosis of either respiratory or cardiovascular disease during follow-up. Subjects were allocated to the ‘Pulmonary’ or ‘Cardiovascular’ group either if they had a history of chronic lung or cardiovascular disease or if a diagnosis of chronic respiratory or cardiovascular disease was made during follow-up. Follow-up was performed by the retrospective review of medical records a mean 27 ± 8 months after CPET. Specifically, charts were reviewed for the following diagnoses: asthma, COPD, pulmonary hypertension, pulmonary embolism, interstitial lung disease, heart failure, coronary heart disease, and dysrhythmias. Subjects with both cardiovascular and respiratory disease were excluded. Ethics committee approval was obtained (CEERB Paris Nord review board).

### Study groups

A total of 77 subjects were included. Thirty had a history of chronic pulmonary disease. Among these, COPD (13 subjects) and asthma (9 subjects) were the most frequent diagnoses. Three subjects had interstitial lung disease, two subjects had a history of surgical lung resection, one had phrenic nerve palsy, one had right-to-left shunting, and one had pleural plaques. Twelve subjects had a history of cardiovascular disease. The most frequent diagnoses were difficult-to-treat hypertension (four subjects), coronary heart disease (three subjects), valvular heart disease (three subjects), and pulmonary embolism (two subjects). None had systolic or diastolic left ventricle dysfunction as assessed by echocardiography.

Among the 35 subjects without a prior history of cardiopulmonary disease, one patient was diagnosed with asthma and was allocated to the ‘Pulmonary’ group. One subject was diagnosed with coronary heart disease, and one subject was diagnosed with paroxysmal atrial fibrillation; both were allocated to the ‘Cardiovascular’ group. The ‘No Disease’ group, thus, comprised 32 subjects, the ‘Pulmonary’ group comprised 31 subjects, and the ‘Cardiovascular’ group comprised 14 subjects.

### Lung function testing

All subjects underwent pulmonary function testing with Jaeger Masterscreen systems (Jaeger, Germany), according to European Respiratory Society guidelines [14]. Tests included slow and forced spirometry, measurement of lung volumes by plethysmography, and single-breath determination of DLCO. Results were considered normal if they were within the range of normal values as determined by European Coal and Steel Community reference equations [15,16]. Chronic dyspnea was rated using the mMRC scale ranging from 0 to 4 [17].

### CPET

Symptom-limited incremental ramp exercise tests were performed according to ATS guidelines using a Masterscreen CPX metabolic cart and a Viasprint cycle ergometer (Carefusion, San Diego, CA) [1]. All tests consisted of a 3-min rest stage, a 2-min warm-up stage at 20 W, an incremental work period at a slope of 10–20 W/min rate, and a 3-min recovery stage. The work rate was selected according to predicted peak power in order to achieve an approximately 10-min work stage. Exercise tests were terminated at the point of symptom limitation or in the presence of electrocardiographic changes consistent with coronary artery disease.

Pulse oxymetry, 12-lead electrocardiography and non-invasive blood pressure measurements were monitored throughout exercise. Breath-by-breath spirometry and metabolic data were collected through a mask. The absence of air leaks was checked by asking the patient to exhale while the airway was obstructed. Minute ventilation, oxygen uptake (VO_2_), carbon dioxide production (VCO_2_), end-tidal CO_2_ partial pressure, tidal volume, and breathing rate were recorded. Oxygen pulse (VO_2_/heart rate) was calculated. The first ventilatory threshold (VT1) was determined by the V slope or VE/VO_2_ nadir methods at the discretion of the attending physician. Slopes of VE/VCO_2_ were determined at VT1 and at peak exercise. Arterial gas was sampled by radial puncture at rest and at peak exercise for blood gas analysis (ABL 725, Radiometer, Copenhagen, Denmark). Physiological dead space (VD/Vt) was calculated by the Bohr–Enghoff equation. The predicted maximal voluntary ventilation was calculated as 35 × forced expiratory volume in 1 s (FEV1) [18]. Ventilatory reserve (VR) was expressed as a percentage of predicted maximal voluntary ventilation. The half-time of VO_2_ recovery (T_1/2_VO_2_) was determined as described [19]. The cardiovascular double product (peak HR × peak systolic blood pressure) was calculated. Predicted values were calculated according to the reference equations of Wassermann [8].

### Statistical analysis

Data were presented as means ± standard deviation. Groups were compared with ANOVA followed by Fisher’s least significant difference test for continuous variables when appropriate, and with the chi-square test for categorical variables. Correlations were examined by linear regression. Statistical analysis was performed with Statview 5.0 (SAS Institute, Cary, NC). To determine the diagnostic performance of CPET parameters for the discrimination of subjects with or without organic heart/lung disease, receiver operating characteristic (ROC) curves were constructed for parameters which were statistically different between groups using the JROCFit tool [20]. ROC curves were compared using the Vassarstats tool (URL: http://vassarstats.net/roc_comp.html) [21]. Youden’s index (sensitivity + specificity –1) was calculated; the highest value was considered the optimal cutoff value. Confidence intervals for sensitivity and specificity were calculated with Vassarstats.

## Results

### Subjects characteristics

Resting characteristics of patient groups are described in Table 1. The groups were similar in terms of age, sex, and body mass index (BMI). In the Pulmonary group, FEV1 ranged from 60 to 120% of the predicted value, and DLCO ranged from 38 to 94% of the predicted. The Pulmonary group had significantly lower DLCO in comparison with the No Disease group, while there was also a trend toward lower FEV1. Resting characteristics were not different between the Pulmonary and Cardiovascular groups.

**Table 1.** Resting characteristics of subjects with and without pulmonary or cardiovascular disease. The Pulmonary and Cardiovascular groups were compared with the ‘No Disease’ group with Fisher’s least significant difference test when the ANOVA *p*-value was < .05. Results are numbers (%) for categorical variables and mean ± standard deviation for continuous variables.

		No Disease (*n* = 32)	Pulmonary (*n* = 31)		Cardiovascular (*n* = 14)		
Categorical variables	Chi^2^						
Sex (male)	.21	11 (30%)	12 (39%)		7 (50%)		
mMRC dyspnea scale (1/2)	.22	20/12	12/19		6/8		
Continuous variables	ANOVA			p vs No Disease		p vs No Disease	p vs Pulmonary
Age (years)	.21	53±15	54±13		65±16		
BMI	.15	27±4	26±5		29±5		
Smoking (pack-years)	.51	14±15	17±18		10±17		
FEV1 (% predicted)	.08	100±17	88±19		89±17		
DLCO (% predicted)	.002	82+/-12	69±16	0.0005	77±16	.37	.94
RV (% predicted)	.28	105±20	122±39		107±27		
HR at rest (min^−1^)	.18	80±10	84±12		76±10		
Systolic BP at rest (mmHg)	.93	117±18	116±17		118±-19		
Resting PaO_2_ (mmHg)	.22	90±9	83±11		75±24		
Resting PaCO_2_ (mmHg)	.29	40±3	39±3		37±3		

We tested whether CPET variables were different in subjects with pulmonary disease or cardiovascular disease in comparison with subjects without disease. As shown in Table 2, the VR was significantly lower in subjects with Pulmonary disease in comparison with the No Disease group, while items related to the cardiovascular response (peak heart rate, cardiovascular double product) were lower in subjects with Cardiovascular disease in comparison with both the No Disease group and the Pulmonary group, possibly owing to heart rate reducing medication. Items related to ventilatory efficiency (VE/VCO_2_, VD/Vt) were higher in subjects with both Pulmonary and Cardiovascular diseases in comparison with the No Disease group. VD/Vt at peak exercise showed the most significant association with both Pulmonary and Cardiovascular diseases. Neither peak VO_2_, nor oxygen pulse, and nor arterial blood gases at peak exercise were significantly different between groups. When considered apart from other subjects, subjects with asthma were characterized by a trend toward increased VD/Vt at peak exercise (31±13, *p* = .08) in comparison with the No Disease group. The VE/VCO_2_ slope and VD/Vt were correlated, as shown in Table 3, although correlation was not strong. Among patients without disease, three had PaCO_2_ < 35 mmHg at peak exercise, including one < 30 mmHg.

**Table 2.** CPET characteristics of the subjects. The Pulmonary and Cardiovascular groups were compared with the ‘No Disease’ group with Fisher’s least significant difference test when the ANOVA *p*-value was standard deviation.

	ANOVA *p*-value	No Disease (*n* = 32)	Pulmonary (*n* = 31)			Cardiovascular (*n* = 14)			
		Values	Values	p vs No Disease	AUC vs No Disease	Values	p vs No Disease	AUC vs No Disease	p vs Pulmonary
HR at peak exercise (min^−1^)	.008	148±25	141±21	.38		115±22	.002	.775	.013
Peak systolic BP (mmHg)	.11	165±25	166±28			165±34			
Double product (mmHg.Min^−1^)	.003	24528±-5608	23706±5715	.98		19598±5122	.002	.633	.002
Peak VO_2_ (% predicted)	.52	89±18	85±22			78±26			
Peak O_2_ pulse (% predicted)	.86	97±19	97±18			97±31			
VE/VCO_2_ slope at VT1	.01	28±4	34±5	.004	.819	33±5	.06		.54
VE/VCO_2_ slope at peak exercise	.004	34±9	38±6	.001	.729	38±9	.07		.26
VD/Vt at rest (%)	.006	26±8	33±9	.002	.706	33±6	.11		.21
VD/Vt at peak exercise (%)	.0008	25±5	35±10	.0002	.835	34±7	.03	.9	.22
SpO_2_ at peak exercise (%)	.17	98±2	97±4			98±1			
VR (%)	.01	36±15	23±21	.003	.645	38±14	.47		.06
PaO_2_ at peak exercise (mmHg)	.11	101±9	89±18			102±11			
PaCO_2_ at peak exercise (mmHg)	.80	36±4	37±5			35±6

**Table 3.** Linear regression of CPET variables. *p*-values and *R*^2^ are shown.

	Peak VO2 (% predicted)	VE/VCO_2_ slope at AT	VE/VCO_2_ slope at peak exercise	VD/Vt at peak exercise (%)
VR (%)	*p* = .04 *R*^2^ = .054	*p* = .0004 *R*^2^ = .194	*p* = .0001 *R*^2^ = .182	*p* = .001 *R*^2^ = .222
VD/Vt at peak exercise (%)	*p* = .034 *R*^2^ = .1	*p* < .0001 *R*^2^ = .482	*p* < .0001 *R*^2^ = .455	
VE/VCO_2_ slope at peak exercise	*p* = .005 *R*^2^ = .101	*p* < .0001 *R*^2^ = .657		
VE/VCO_2_ slope at AT	*p* = .36 *R*^2^ = .014			

### Diagnostic performance of CPET variables for the diagnosis of pulmonary or cardiovascular disease

ROC curves were constructed for variables that were statistically different between subjects without disease and subjects with pulmonary or cardiovascular disease (Figure 1). The area under the curve (AUC) is shown in Table 2. VD/Vt measured at peak exercise had the highest AUC for the diagnosis of both pulmonary and cardiovascular diseases. In patients with pulmonary disease, the AUC for VD/Vt at peak exercise was significantly greater than the AUC for VR (*p* = .018), and it was not different to the AUC for peak heart rate (*p* = .14). In patients with pulmonary disease, the AUC for VD/Vt at peak exercise was significantly greater than the AUC for VR (*p* = .026), and it was not different to the AUC for VE/VCO_2_ at VT1, VE/VCO_2_ at peak exercise, and VD/Vt at rest (*p* = .43, .12, and .09, respectively). Youden’s index was maximal at a threshold of 28%, where the sensitivity and specificity of VD/Vt at peak exercise for discriminating subjects without disease from subjects with either pulmonary or cardiovascular disease were 89% (95% CI: 64–98%) and 72% (95% CI: 46–89%).

## Discussion

The main result of this study is that VD/Vt at peak exercise was the CPET variable most related to both mild pulmonary and cardiovascular disease in subjects with mild-to-moderate dyspnea. At a threshold of 28%, VD/Vt had a sensitivity of 89% and a specificity of 72% for cardiopulmonary disease. This finding is consistent with the notion that a majority of cardiac and pulmonary disorders affect either the ventilatory pattern resulting in increased anatomical dead space ventilation or pulmonary ventilation/perfusion matching resulting in alveolar dead space ventilation. Ventilatory inefficiency is a powerful marker of severity in a wide range of cardiopulmonary diseases including heart failure [22,23], COPD [24], interstitial lung diseases [25], and pulmonary hypertension [26,27], in terms of both survival and functional limitation. Our results suggest that increased VD/Vt may be a physiological marker of mild heart or lung disease. In support of this hypothesis, increased VD/Vt is the most frequent CPET finding in asymptomatic patients with surveillance-detected preclinical beryllium disease [28]. This concept may, in part, be relevant to airway diseases, since a trend toward increased VD/Vt was observed in subjects with asthma, consistent with previous studies [29,30, 31].

The notion that CPET as a whole is sensitive for diagnosing an organic cause to exercise limitation has been supported by previous authors [32,33]. Our observation that pulmonary or cardiovascular disease was subsequently diagnosed in only 3 out of 35 subjects without such prior history left out 32 subjects without a diagnosis for their complaint of dyspnea, raising the concern that subsequent diagnoses may have been underreported. In this regard, a limitation of the present study is that the absence of disease was defined as the lack of a recorded diagnosis of cardiopulmonary disease in follow-up outpatient charts, thus with potential attrition bias. Reporting bias is also a possible limitation of the study. For instance, mild or early heart failure with preserved ejection fraction [34] or neuromuscular disease [35] would be possible difficult diagnoses. This caveat aside, the observation that no organic disease was detected in 32 out of 35 previously healthy subjects with a complaint of mild or moderate dyspnea is consistent with a previous report where no heart or lung disease was evidenced in 31 out of 50 dyspnoeic patients [32]. Although we did not consider such diagnoses in the absence of consensual diagnostic criteria [36], it is conceivable that either poor conditioning or psychogenic dyspnea such as that observed in the hyperventilation/dysfunctional breathing syndrome or medically unexplained dyspnea [37], conditions in which subjects may be hypersensitive to the unpleasantness of breathlessness [38], may have explained symptoms in some patients. Neither poor conditioning nor psychogenic dyspnea is expected to alter pulmonary gas exchange. Low VD/Vt values may help with establishing such diagnoses.

Several elements related to study design and technical issues warrant caution regarding interpretation of the present results. It is probable that the inclusion of an asymptomatic control group would have strengthened our results. Subjects with chronic lung disease were overrepresented compared with subjects with heart disease. Applicability of our findings is uncertain due to the heterogeneity of the study population. Subjects underwent neither NT-BNP testing which may be of use for the diagnosis of heart failure, nor right heart catheterization, nor assessment of respiratory muscle strength, nor measurements of respiratory drive. Thus, it is possible that some patients in the No Disease group were misdiagnosed. It is possible that heart-slowing drugs may have altered heart rate and cardiovascular double product in the Cardiovascular group. Differing levels of physical activity may also have contributed to the differences observed between groups.

VD/Vt values in the No Disease group were high in comparison with previous reports [39]. Although the PaCO_2_ kinetics immediately following maximal incremental exercise are not known, in a previous study PaCO_2_ increased in 25 of 137 subjects following stair climbing exercise [40]. Thus, we suspect that slight asynchrony between the moment mean expired CO_2_ was determined (i.e. at peak exercise) and the moment when arterial blood was obtained for determination of PaCO_2_ (i.e. from a few seconds to 1 min following exercise cessation) may explain both the high VD/Vt values at peak exercise in subjects without disease, and the lack of a significant decrease in VD/Vt at exercise in all groups. We acknowledge that rather than arterial puncture, measurement of capillary blood gases or insertion of an arterial catheter for arterial blood gas analysis would have allowed better time matching of gas and blood samples and thus more accurate determination of physiological VD/Vt. However, these techniques were not available in the context of a retrospective study. Likewise, technical limitations did not allow the use of techniques other than the Bohr–Enghoff equation for the determination of dead space ventilation. Maximal voluntary ventilation was not measured but estimated by multiplying FEV1; thus; VR may have been overestimated in some subjects. Static and mechanical constraints, such as dynamic overinflation [3], and their relationships with ventilatory efficiency were not studied. Overall, these limitations possibly resulted in underestimation of the diagnostic performance of VE/CO_2_ and VD/Vt measurements.

We believe that a key strength of this study was the inclusion of patients without severe abnormalities of resting tests. Indeed, although DLCO was different between the subjects without Pulmonary or Cardiovascular disease, and subjects with No Disease, there was considerable overlap precluding the use of lung function tests to reliably rule out disease. The patient mix reflected the real-world situations, where CPET is used for the differential diagnosis of exertional dyspnea when first-line tests fail to detect severe pulmonary or cardiovascular disease, and thus allowed us to assess its diagnostic performance to distinguish patients with mild disease from subjects without disease.

From a clinical perspective, our results suggest that analysis of parameters related to ventilatory efficiency, i.e. physiological VD/Vt at exercise or VE/VCO_2_ at VT1, is sensitive for ruling out organic cardiac or pulmonary disease in subjects with mild dyspnea, in line with a previous study where a normal VE/VCO_2_ at VT1 reliably predicted the absence of pulmonary embolism [41]. This information may be useful for the interpretation of CPET, with the caveat that although significant differences were found in VE/VCO_2_ and VD/Vt between groups of subjects with or without organic disease, there was significant overlap, resulting in limited sensitivity and specificity and mandating caution for their use in individual patients. Although interpretation of CPET does not rest on a single parameter but instead lies in the integration of multiple data including the results of resting tests, better knowledge of the diagnostic information provided by each individual measurement may increase the overall performance of CPET interpretation. Integration of the full spectrum of clinical, physiological, or imaging data for the exploration of dyspnea was beyond the scope of this study.

## Conclusions

While CPET is recognized as a worthy component of the diagnostic arsenal for the exploration of dyspnea, questions remain as to its optimal place in diagnostic strategies. Our results indicate that increased physiological VD/Vt at exercise is a sensitive and specific marker of mild pulmonary or cardiovascular disease in dyspneic subjects.

## Statement

The authors state that the manuscript has been read and approved by all the authors, that the requirements for authorship have been met, and that each author believes that the manuscript represents honest work.
